# The Book World of Medicine and Science

**Published:** 1900-12-15

**Authors:** 


### THE BOOK WORLD OF MEDICINE AND SCIENCE.

Twentieth Century Practice: An International Encyclopædia of Modern Medical Science by Leading Authorities of Europe and America. Edited by Thomas L. Stedman, M.D. Volume XX. (London: Sampson Low, Marston and Co. 1900.)

Dr. Stedman is to be congratulated on having now completed this great encyclopædic work. He has fully performed the promises which were held out at the commencement of his labours, and no one who studies the volumes which have appeared so punctually one after the other can have any doubt as to the excellence of the series of monographs which they contain on the various subjects included in the wide title "Modern Medical Science." As was inevitable, all these essays have not attained quite the same standard, but they have all expressed the ideas and the methods of men who are recognised authorities on the topics with which they have dealt, and it may fairly be said that these twenty volumes represent in a most satisfactory manner the medicine of to-day. The volume now before us contains articles on Tuberculosis, Yellow Fever, Poisoning with Snake Venom, Mushroom Poisoning, Diseases of the Uvula, Soft Palate and Fauces, and Neural and Mental Defects in Childhood—somewhat of a hotch-potch, truly, but essential for completeness.

Dr. August Jerome Lartigau, of New York, deals with the bacteriology, pathology, and etiology of tuberculosis in an interesting essay of 140 pages. On the question of the relationship between the tubercle bacilli from different sources he says that the unity of the bacillus tuberculosis of mammals has now been established, but that the question whether the human, i.e. the mammalian, bacillus is identical with that met with in birds still remains under discussion. He thinks, however, that we are now near the solution of this heretofore much vexed question, for the investigations of Bataillon, Terre, and Dubard having been verified, it seems clear that the unity of all tubercle bacilli from whatever source has been established; variations being merely accidents of long habit in certain environments. It has now been shown that human and even avian bacilli, with the establishment of adequate conditions, may induce tuberculous lesions in cold-blooded animals, such as frogs, fishes, &c.

In the article on symptomatology, by Dr. Henry W. Berg, of New York, a very good description of the disease in its several varieties is to be found. There is no doubt, however, that the most important article of the series, and indeed the most important in the whole volume, is that by Dr. S. A. Knopf, of New York, which is concerned principally with treatment and proplylaxis. In this article we find a full and graphic account of the methods which Dr. Knopf has proved to be useful in his own experience, as well as of those which others have employed. The sanatorium treatment of consumption is described in all its details, and many plans and illustrations are given. Dr. Knopf says that this is the line of treatment which has so far proved the most successful, and that it possesses the additional advantage that it can be carried out in all climates. In regard to the latter point he instances the Victoria Hospital at Edinburgh as one in which most satisfactory results are obtained, saying that "what is possible in Scotland with its rigorous climate is possible anywhere else," and he adds that he knows of two institutions in the United States, located but a few miles from two of the largest cities, the results obtained at which are certainly most remarkable, although no special climatic advantages can be claimed for either of them. We confess that after all this we are a little surprised to find one who is so enthusiastic about open air, and who has done so much to bring the open-air treatment into practical use, should speak so highly as he does of treatment by the "pneumatic cabinet." However, we must take his word for it.

The subject of yellow fever is carefully, although not very convincingly, treated by Dr. Wolfred Nelson. The history of the disease is entered into pretty fully, and a very complete description is given of the methods of quarantine and disinfection adopted at New Orleans to prevent the disease from gaining entry into the territory of the United States. In regard to the etiology and pathology of the disease, however, Dr. Nelson's treatise is hardly so satisfactory as might have been desired. The last article in the volume is one by Dr. Francis Warner, of London, on neural and mental defects in childhood, in which he gives a good account of the results of his well-known researches on this subject. Finally we come to a full and admirable general index to the whole twenty volumes. This is a very praiseworthy performance, and forms a fitting termination to a work the preparation and editing of which must have cost Dr. Stedman an enormous amount of labour, and on the punctual and satisfactory completion of which he deserves hearty congratulation. It has been an arduous task and it has been well performed.

China and the Present Crisis. By R. L. Jefferson, F.R.G.S. With map and glossary. (London: G. W. Bacon and Co. 1900. Price 3d.)

This is a little pamphlet, with a small map, which gives a good deal of useful information about China and its people. To those who possess much knowledge on these subjects this pamphlet will probably appear trivial and superficial, as indeed it is. Still, there are many thousands whose ideas about China are absolutely vague, and to them this threepennyworth of knowledge should be a good investment.

---

### NOTES ON BOOKS.

Bunce, the Bobby, and the Broads: a Holiday Yarn, by Fritz Zorn (Jarrold and Sons), tells how "I" went for a holiday cruise on the Norfolk Broads accompanied by a policeman—bent on the same quest—and Bunce, a previous acquaintance. The story is told in an amusing and easy style. The descriptions of the ins and outs of the Broads is good, though the local colouring and geography act chiefly as a background for the jokes and racy conversation of the Bobby, who, to use his own phraseology, is a "fly" one. A book only for a *very* idle hour.

